# Comparing the Effectiveness of 0.12% Chlorhexidine and Silver Nanoparticles Mouthwashes: A Double-Blind Randomized Controlled Trial Among Medical Students in Southern Vietnam

**DOI:** 10.7759/cureus.87948

**Published:** 2025-07-14

**Authors:** Do T Thao, Yen H Nguyen Thi, Duy H Le Nguyen, Anh T Dang The, Cuong P Tru Nguyen, Vy T Nguyen, Uyen N Quach, Loc T Truong

**Affiliations:** 1 Oral Pathology and Periodontology Department, Faculty of Odonto-Stomatology, Can Tho University of Medicine and Pharmacy, Can Tho, VNM; 2 Microbiology Department, Faculty of Medicine, Can Tho University of Medicine and Pharmacy, Can Tho, VNM; 3 Dentistry Department, Faculty of Odonto-Stomatology, Can Tho University of Medicine and Pharmacy, Can Tho, VNM

**Keywords:** chlorhexidine, dental plaque, gingivitis, mouthwash, preventive dentistry, silver nanoparticles

## Abstract

Background: Dental plaque and gingivitis are prevalent conditions that can be controlled using antimicrobial mouthwashes.

Aim and design: This double-blind, randomized controlled clinical trial aims to compare the effectiveness of 0.12% chlorhexidine (CHX) and silver nanoparticles (AgNPs) mouthwashes in managing dental plaque, gingivitis, microbial counts, and saliva pH.

Materials and methods: A total of 95 medical students were randomly divided into three groups: Group I (control group), 0.9% sodium chloride; Group II, 0.12% CHX mouthwash; and Group III, AgNPs mouthwash. Clinical parameters evaluated on the baseline and 21stday included the gingival index, Quigley‐Hein plaque index (QHI), microbial counts, and saliva pH. The analysis of variance (ANOVA), Tukey post-hoc test, and paired t-tests were implemented.

Results: The QHI in Group II decreased from 2.66 ± 0.75 to 1.97 ± 0.61 after 21 days (p < 0.001). Specifically for the QHI, there is a statistically significant difference between Group II and Group III, while there is no statistically significant difference when comparing the two groups in other clinical indices, and both groups showed a noticeable improvement after 21 days of use.

Conclusions: Both 0.12% CHX and AgNPs mouthwashes are appropriate alternative options for daily oral hygiene. However, CHX mouthwash should still be prioritized for plaque control.

## Introduction

The oral biofilm is a habitat for numerous bacterial species that cause various oral diseases, such as dental caries and periodontal diseases [1]. These biofilm-related conditions continue to pose significant challenges for dental professionals [2]. Controlling dental biofilm is crucial in managing dental caries, and this has traditionally been achieved primarily through mechanical methods [3]. As an adjunct that provides benefits beyond mechanical methods, mouthwashes have been employed as antimicrobial agents in dental biofilm control [4]. Antimicrobial mouthwashes can reduce the microbial counts in the oral cavity, alleviating oral diseases and various inflammatory conditions [5].

Chlorhexidine (CHX) is a common antibacterial mouth rinse in dentistry. CHX can adhere to oral tissues, providing antibacterial effects for a fixed period [5,6]. However, the prolonged use of CHX may lead to adverse effects, which have been evaluated and include dark staining of teeth, chemical interactions with sodium hypochlorite resulting in the formation of flocculation, biological hazards, and interactions with filling materials [7]. Additionally, its unpleasant taste and the potential for resistance in oral bacteria, as well as cross-resistance to antibiotics, are other drawbacks associated with CHX [4].

Consequently, researchers have been exploring alternative antibacterial agents for use as mouthwashes [8]. In recent years, we have seen the integration of nanotechnology into various scientific sectors, as it provides solutions to several technological and medical problems [9]. Among the metals with antibacterial properties, silver has a strong antibacterial effect [10,11]. Metal nanoparticles exhibit specific biochemical and physical properties when used to combat pathogenic microorganisms [12]. Silver nanoparticles (AgNPs) mouthwash has strong antibacterial properties, as shown in recent trials [1,5,13].

Previous studies have compared the effectiveness of CHX and AgNPs mouthwashes in rabbits [5] and humans [14], and their antibacterial effects resist various bacterial strains [15,16]. Furthermore, in vitro research comparing CHX and AgNPs mouthwashes has been documented [3,17]. Nevertheless, these studies present limitations in that they have investigated only a few species of bacteria, were conducted in an artificial oral environment, and included no statistically significant sample sizes large enough to make meaningful comparisons [5,14].

This study aimed to evaluate and compare the effectiveness of 0.12% CHX and AgNPs mouthwashes in managing gingival inflammation, dental plaque, antimicrobial activity, and changes in saliva pH, in comparison with a control group, among students of Can Tho University of Medicine and Pharmacy during the 2023-2024 academic year.

## Materials and methods

Study participants

A randomized, double-blind, controlled clinical trial was conducted at the Faculty of Odonto-Stomatology, Can Tho University of Medicine and Pharmacy Hospital, Can Tho, Vietnam. The study followed a parallel group design allocation ratio and a superiority framework to compare the effectiveness of three different mouthwashes over 21 days. The study was carried out after receiving approval from the Ethics Committee of Can Tho University of Medicine and Pharmacy on February 6th, 2024 (23.134.SV/PCT-HĐĐĐ). In addition, the trial was registered on ClinicalTrials.gov under the identifier NCT06963788. All participants were fully informed about the content and procedure of the study, and were informed to comply with the instructions and complete the research volunteer consent form. No changes were made to the protocol after trial commencement. Students from Can Tho University of Medicine and Pharmacy in the 2023-2024 academic year were informed about the research and volunteered to fill in the information to participate in the research via a Google Form.

Test products

The study employed three products for evaluation: the 0.9% sodium chloride (NaCl); the 0.12% CHX mouthwash containing purified water, CHX gluconate, polyethylene glycol 40 (PEG-40), and sodium citrate; the AgNPs 7 ppm mouthwash including purified water, nano silver, sodium bicarbonate, sodium borate, sodium benzoate, and hydrogenated polyethylene glycol 40.

Study design

The research has the following inclusion criteria: participants using no systemic antibiotics, corticosteroids, or mouthwash for at least 28 days before the study and maintaining stable oral and general health [16,18]. Exclusion criteria for the study were participants with incomplete information or lack of cooperation; who have an oral infection; ongoing orthodontic or oral aesthetic treatments; and systemic diseases such as autoimmune disorders, diabetes, hypertension, and hematological or psychiatric conditions [16].

We invited 98 medical students to voluntarily participate in this double-blind interventional study, which included randomized controlled trials, conducted from February to March 2024 in the Faculty of Odonto Stomatology, Can Tho University of Medicine and Pharmacy. During the course of the study, three participants were excluded based on the exclusion criteria. The final sample size of the study was 95 participants.

Study participants were randomly assigned to three study groups through a lottery method. Individuals were identified by code throughout the study. Group I used 0.9% NaCl, Group II used 0.12% CHX mouthwash, and Group III used AgNPs mouthwash.

The random allocation sequence was generated by Investigator A using a simple lottery method. To ensure allocation concealment, 99 folded papers (33 labeled Number 1, 33 labeled Number 2, and 33 labeled Number 3) were placed in sealed opaque envelopes and shuffled thoroughly. Each participant randomly selected one envelope to determine group assignment. Participants who drew paper Number 1 were assigned to Group I (NaCl 0.9%), Number 2 to Group II (CHX 0.12%), and Number 3 to Group III (AgNPs). This method ensured equal allocation probability while maintaining allocation concealment. Investigator A, who was not involved in outcome assessment or data analysis, managed the randomization process. Investigator B, responsible for clinical assessment and data analysis, was fully blinded to the group allocations and was unaware of which type of mouthwash each participant received [19].

**Figure 1:**
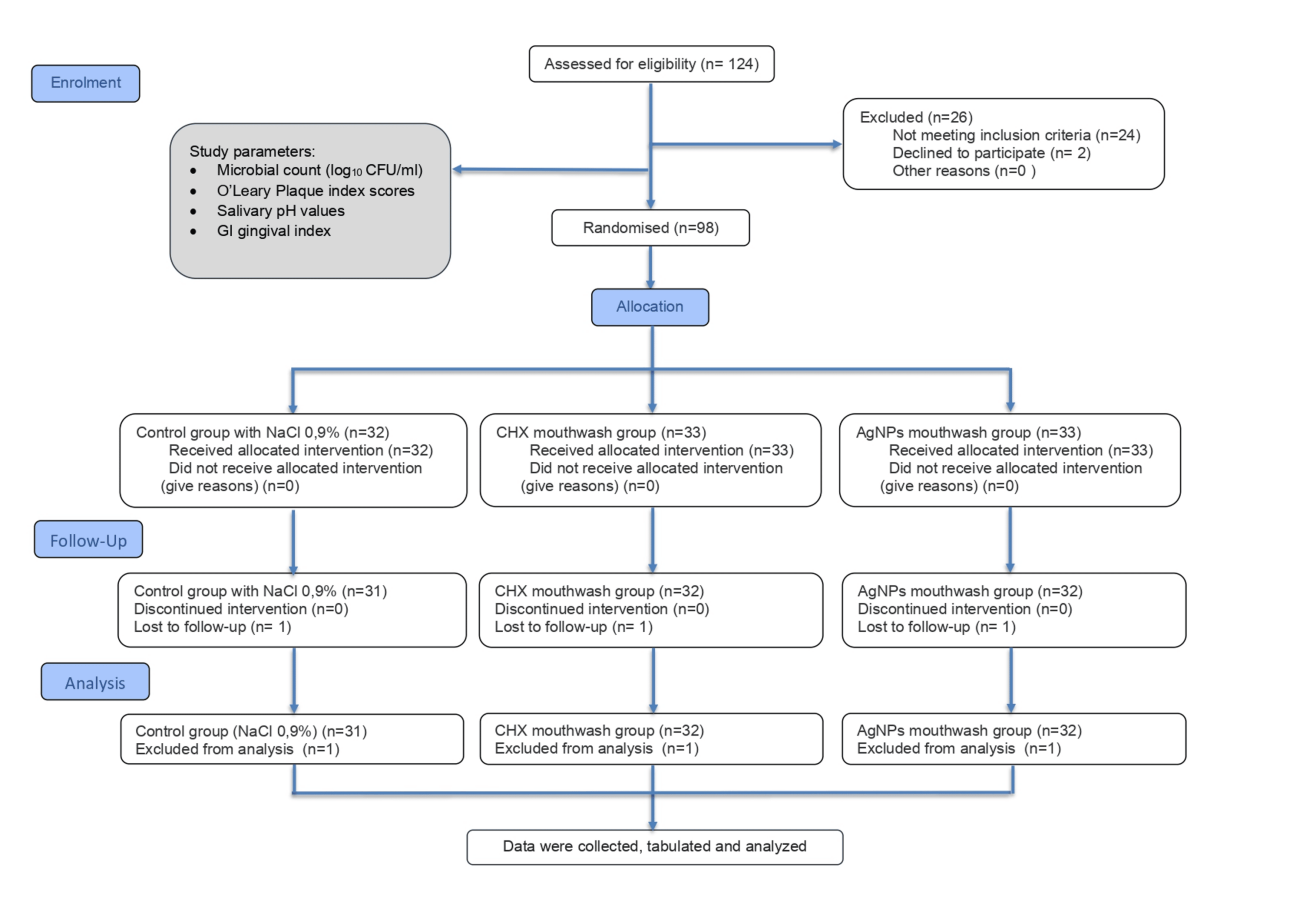
The Consolidated Standards of Reporting Trials (CONSORT) flow diagram of the study recruitment. CFU: colony-forming unit; Group I (control group): 0.9% sodium chloride (NaCl); Group II: 0.12% chlorhexidine (CHX) mouthwash; Group III: silver nanoparticles (AgNPs) mouthwash

Clinical parameters obtained at baseline (before the research) and after 21 days (at the end of the research) included the gingival index (GI) according to Loe (1967) [20], the Quigley-Hein plaque index (QHI) [21], microbial counts, and saliva potential of hydrogen (pH) [16]. The Consolidated Standards of Reporting Trials (CONSORT) flow diagram illustrating the study recruitment process is presented in Figure 1.

Sample collection procedure

Before the study, participants underwent a comprehensive oral health screening to evaluate dental caries, inflammation, and other dental conditions. Participants were instructed to fast for two hours prior to the examination to ensure accurate results. The selection was based on specific inclusion and exclusion criteria, and baseline clinical characteristics were accurately recorded.

All participants were instructed to use 10 mL of the assigned mouthwash twice daily after brushing, rinsing for 30 seconds each time, over a period of 21 days. A designated monitoring team was responsible for reminding participants to adhere strictly to the protocol, and participants were required to commit to compliance.

**Figure 2:**
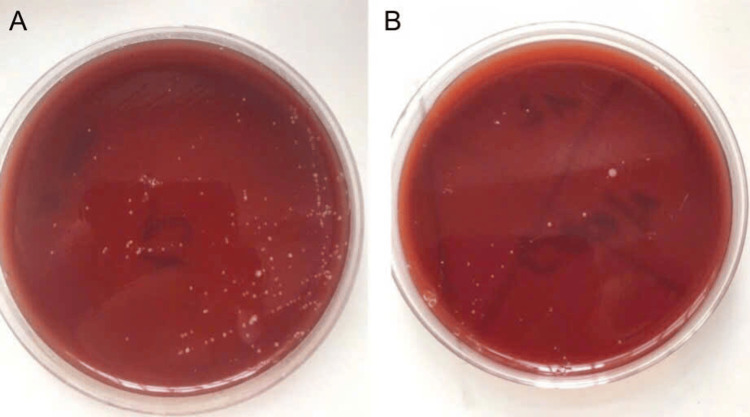
Microbial count on blood agar. (A) Baseline; (B) On the 21st day following the use of silver nanoparticles mouthwash.

**Figure 3:**
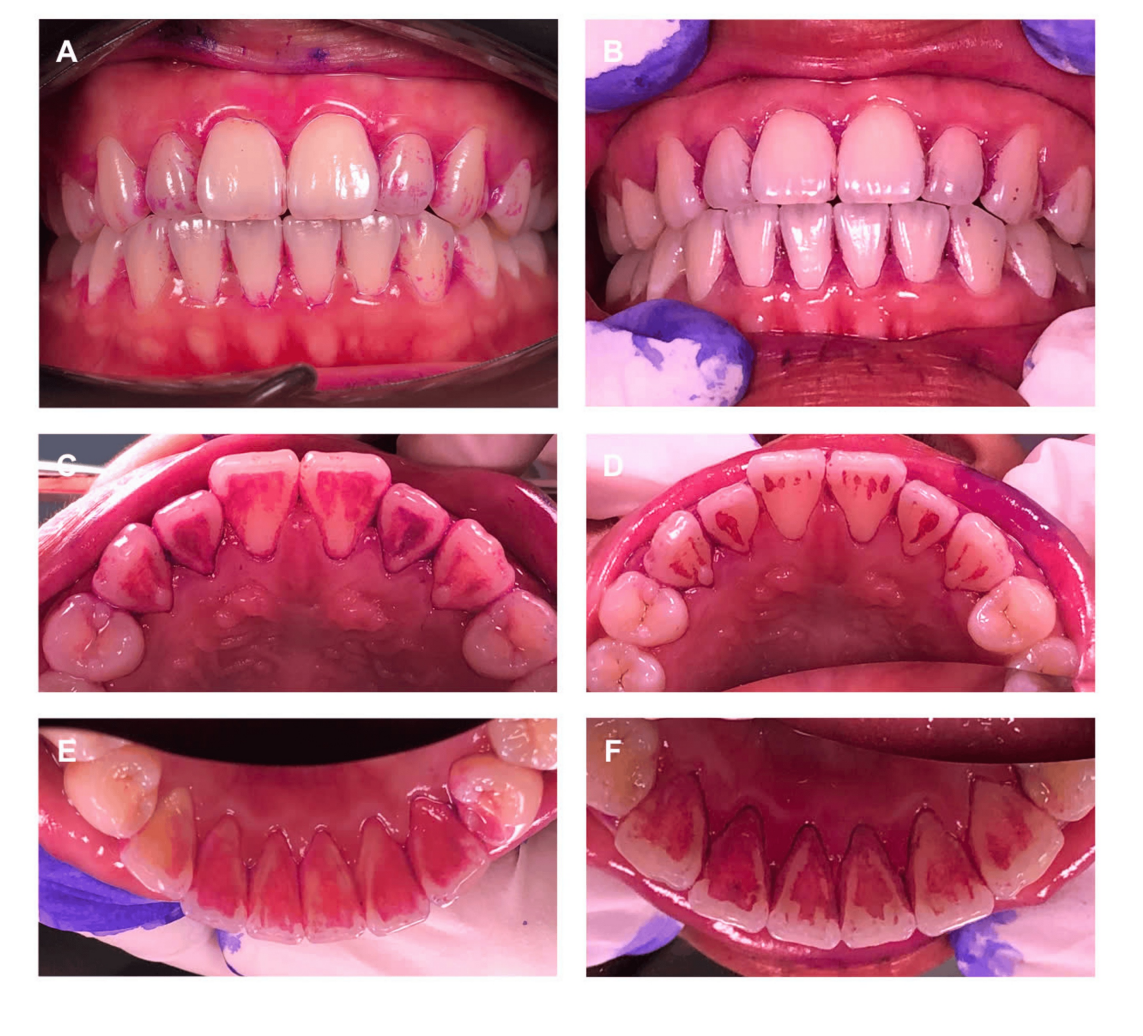
Dental plaque after the application of disclosing tablets. (A) The facial surface observed on the baseline day; (B) The facial surface observed on day 21 after using silver nanoparticles mouthwash; (C) The palatal surface of the maxilla observed on the baseline day; (D) The palatal surface of the maxilla observed on day 21 after using silver nanoparticles mouthwash; (E) The lingual surface of the mandibular observed on the baseline day; (F) The lingual surface of the mandibular observed on day 21 after using silver nanoparticles mouthwash.

At baseline (day 0), a detailed assessment of microbial counts was conducted. Saliva samples were cultured on agar plates with standardized volumes of 10 µL, diluted to concentrations of 1/100 and 1/1000. After incubation at 37°C for 24 hours, colony-forming units (CFUs) were counted, and the microbial density was calculated per milliliter of saliva, then converted to a logarithm (CFU/mL) for statistical analysis. Additionally, saliva pH was measured using a calibrated digital pH meter, equipped with an electrode and a temperature sensor (Hanna HI2211-02 Benchtop pH Meter, Italy). The plaque level was assessed by using the QHI. After 21 days of use (day 21), a reassessment was conducted to reflect the processes from day 0, including clinical parameters such as GI, QHI, microbial count, and salivary pH (Figures 2-3).

Statistical analysis

The assembled data were imported into Microsoft Excel 2020 (Microsoft® Corp., Redmond, WA, USA) and Google Drive (Google, Inc., Mountain View, CA, USA), and subsequently analyzed using IBM SPSS Statistics for Windows, Version 22 (Released 2015; IBM Corp., Armonk, New York, United States). The analysis of variance (ANOVA), paired t-tests, and Tukey post-hoc tests were employed to evaluate study parameters at baseline and after 21 days of mouthwash use. Three participants were lost to follow-up before the day 21 assessment, resulting in missing outcome data. These participants were excluded from the final analysis. The analysis was conducted on a modified intention-to-treat basis, including only participants with complete data.

Study error

The total number of CFUs was counted and analyzed by a microbiologist. Parameters such as the GI, QHI, and salivary pH were assessed by a dentist. Investigators were blinded to the sample group assignments. The microbiologist's consistency was evaluated as follows: after the initial measurements, 30 randomly selected samples were re-evaluated by the same individual using the same method (reproducibility testing), approximately 30 minutes later. Similarly, the dentist’s intra-rater reliability was assessed using the same approach. Data from the second measurements were compared with the first using Pearson’s correlation coefficient.

## Results

A total of 95 participants, including 36 males (37.89%) and 59 females (62.11%), completed the study. The mean age of the participants was 19.00 ± 1.13.

Regarding microbial counts, a statistically significant difference was observed among the three groups after 21 days of using the trial products. This difference was particularly evident between Group I and Group II, and between Group I and Group III. In contrast, no significant difference was found between Group II and Group III. Furthermore, significant reductions in microbial counts were observed within both Group II and Group III. Specifically, the mean count in Group II decreased from 7.90 ± 0.20 to 7.42 ± 0.51, and in Group III from 7.84 ± 0.29 to 7.33 ± 0.55 (Table 1).

**Table 1:** Mean values and paired comparisons of the microbial count (log10 CFU/mL) at baseline and on the 21st day. SD: standard deviation; Group I: 0.9% sodium chloride; Group II: 0.12% chlorhexidine (CHX) mouthwash; Group III: silver nanoparticles (AgNPs) mouthwash; ANOVA: analysis of variance * p-value < 0.05 is significant.

Day	Paired-sample t-test									ANOVA		Tukey post-hoc tests		
	Group I			Group II			Group III							
	Mean ± SD	t	p-value	Mean ± SD	t	p-value	Mean ± SD	t	p-value	F	p-value	Groups I-II	Groups I-III	Groups II-III
Baseline	7.86 ± 0.24	0.456	0.653	7.90 ± 0.20	5.271	<0.001*	7.84 ± 0.29	5.244	<0.001*	0.517	0.320	0.732	0.977	0.600
21st day	7.84 ± 0.23			7.42 ± 0.51			7.33 ± 0.55			10.383	<0.001*	<0.001*	<0.001*	0.925

In terms of GI, a marked reduction was observed after 21 days in both Group II (from 0.34 ± 0.24 to 0.05 ± 0.03) and Group III (from 0.37 ± 0.21 to 0.06 ± 0.03). Intergroup comparisons also revealed statistically significant differences, especially between Group I and Group II, and between Group I and Group III (Table 2).

**Table 2:** Mean values and paired comparisons of the gingival index at baseline and on the 21st day. SD: standard deviation; Group I: 0.9% sodium chloride; Group II: 0.12% chlorhexidine (CHX) mouthwash; Group III: silver nanoparticles (AgNPs) mouthwash; ANOVA: analysis of variance * p-value < 0.05 is significant.

Day	Paired-sample t-test									ANOVA		Tukey post-hoc tests		
	Group I			Group II			Group III							
	Mean ± SD	t	p-value	Mean ± SD	t	p-value	Mean ± SD	t	p-value	F	p-value	Groups I-II	Groups I-III	Group II-III
Baseline	0.33 ± 0.17	1.492	0.146	0.34 ± 0.24	6.760	<0.001*	0.37 ± 0.21	8.236	<0.001*	0.305	0.738	0.984	0.739	0.833
21st day	0.25 ± 0.24			0.05 ± 0.03			0.06 ± 0.03			21.207	<0.001*	<0.001*	<0.001*	0.981

For salivary pH, significant increases were recorded in both Group II (from 7.18 ± 0.34 to 7.41 ± 0.44) and Group III (from 7.16 ± 0.39 to 7.41 ± 0.38). Intergroup comparison revealed statistically significant differences (p = 0.021), particularly between Group I and Group II, and between Group I and Group III (Table 3).

**Table 3:** Mean values and paired comparisons of saliva pH values at baseline and on the 21st day. SD: standard deviation; Group I: 0.9% sodium chloride; Group II: 0.12% chlorhexidine (CHX) mouthwash; Group III: silver nanoparticles (AgNPs) mouthwash; ANOVA: analysis of variance * p-value < 0.05 is significant.

Day	Paired-sample t-test									ANOVA		Tukey post-hoc tests		
	Group I			Group II			Group III							
	Mean ± SD	t	p-value	Mean ± SD	t	p-value	Mean ± SD	t	p-value	F	p-value	Groups I-II	Groups I-III	Groups II-III
Baseline	7.20 ± 0.38	-0.748	0.460	7.18 ± 0.34	-3.957	<0.001*	7.16 ± 0.39	-3.399	0.002	0.148	0.863	0.946	0.851	0.972
21st day	7.26 ± 0.32			7.41 ± 0.44			7.41 ± 0.38			4.037	0.021*	0.044*	0.037*	0.997

Regarding the QHI, a statistically significant difference was found among the three groups (p = 0.002), particularly between Group I and Group II, and between Group II and Group III. The QHI in Group II showed a notable reduction from 2.66 ± 0.75 to 1.97 ± 0.61. However, no significant decrease in QHI was observed in Group I or Group III compared to baseline values (Table 4).

**Table 4:** Mean values and paired comparisons of the Quigley-Hein plaque index at baseline and on the 21st day. SD: standard deviation; Group I: 0.9% sodium chloride; Group II: 0.12% chlorhexidine (CHX) mouthwash; Group III: silver nanoparticles (AgNPs) mouthwash; ANOVA: analysis of variance * p-value < 0.05 is significant.

Day	Paired-sample t-test									ANOVA		Tukey post-hoc tests		
	Group I			Group II			Group III							
	Mean ± SD	t	p-value	Mean ± SD	t	p-value	Mean ± SD	t	p-value	F	p-value	Groups I-II	Groups I-III	Groups II-III
Baseline	2.64 ± 0.68	0.378	0.708	2.66 ± 0.75	4.847	<0.001*	2.65 ± 0.79	0.949	0.350	0.009	0.991	0.990	0.997	0.998
21st day	2.60 ± 0.80			1.97 ± 0.61			2.52 ± 0.6			8.666	<0.001*	0.001*	0.946	0.002*

## Discussion

Nowadays, CHX mouthwash is still considered the gold standard in the treatment and prevention of oral diseases, a fact that has been demonstrated and recognized in numerous previous studies. Our research also shows similar results, with the group using CHX mouthwash showing significant effectiveness in all study parameters after 21 days of use [3,16,22-24]. This effectiveness is largely attributed to CHX's mechanisms of action, which include its positive charge attracting it to negatively charged bacterial cell walls. Upon interaction with the phospholipids in the bacterial cell membrane, CHX increases membrane permeability and disrupts its structural integrity, leading to the leakage of intracellular contents and eventual bacterial cell death. Additionally, CHX forms a thin film on tissue surfaces, retaining the active ingredient and providing prolonged antimicrobial effectiveness [11,24]. Recent studies on AgNPs, with their superior antibacterial properties, have become a focus of researchers in all relevant fields [25]. Recently, the antibacterial effectiveness of mouthwash containing AgNPs has also shown significant results in clinical trial studies as well as in vitro research [3,16,26]. Our research also shows similar results, AgNPs mouthwash demonstrating significant effectiveness in almost all study parameters, except for QHI. The antibacterial effectiveness of AgNPs may be attributed to several well-established mechanisms. First, AgNPs can adhere to the microbial cell membrane, altering its permeability and structural integrity. Second, they are capable of penetrating bacterial cells, thereby disrupting biomolecular structures and causing significant intracellular damage. Third, AgNPs generate reactive oxygen species (ROS), which induce oxidative stress and lead to cellular dysfunction. Finally, they interfere with bacterial signaling pathways, inhibiting vital cellular processes. Collectively, these mechanisms support the potential of AgNPs as a promising adjunct in improving oral health through effective microbial control [13].

In a study by Al-Sharani et al. comparing the effects of AgNPs and 0.12% CHX mouthwashes on microbial counts in the plaque of patients with plaque-induced gingivitis, it was found that CFU/mL values significantly decreased after four weeks of using the AgNPs mouthwash, with a bacterial growth inhibition rate of up to 79.7%. While the AgNPs mouthwash exhibited a higher degree of bacterial inhibition relative to the CHX mouthwash, statistical analysis revealed no significant difference between the two treatments upon direct comparison [26]. Moaddabi et al. also conducted a study on rabbits, which concluded that there was no significant difference between these mouthwashes. It was also suggested that the 9.80 wt% AgNPs mouthwash, with a particle size of 5 nm, has antibacterial effects and wound-healing properties, making it a promising alternative for use after oral surgical procedures. The results of our study are also consistent with these findings. Both the 0.12% CHX and AgNPs mouthwashes in our study showed significant effectiveness in reducing microbial counts, with no significant difference after 21 days of use [5]. Furthermore, Panpaliya et al. found that AgNPs demonstrated better antibacterial effectiveness than CHX. They suggested that AgNPs exhibit bacteriostatic and bactericidal effects more effectively at concentrations five times lower than CHX. When used at appropriate concentrations, AgNPs represent a safe alternative to other chemical-based antimicrobial agents [3]. However, their in vitro study cannot fully replicate the oral environment, so more clinical trials are needed for further clarification. On the other hand, the effectiveness of AgNPs mouthwash could also be influenced by the size of the AgNPs used in the study, as demonstrated by Espinosa-Cristóbal et al., who showed that the antibacterial activity of AgNPs against *Streptococcus mutans* depends on the size of the particles. Specifically, smaller nanoparticles are capable of releasing more silver ions, leading to a more effective antibacterial effect [27]. Similarly, CHX mouthwash at different concentrations also has varying levels of effectiveness [28].

When comparing the effectiveness between 0.12% CHX and AgNPs mouthwashes, the results of our study regarding gingivitis and salivary pH values are similar to the studies by El Din et al. [14], Al-sharani et al. [29], and Maher et al. [16]. Both El Din et al. and Al-sharani et al., when evaluating the effects of AgNPs and CHX mouthwash on plaque-induced gingivitis, showed no statistically significant difference after 28 days of use. They suggested that AgNPs mouthwash could be an alternative to CHX due to its lack of side effects. However, Richards pointed out that, clinically, the reduction in GI in individuals with mild gingivitis (average GI score from 0.1 to 1) was not significant, and there was insufficient evidence to determine the level of gingivitis reduction associated with CHX mouthwash in individuals with moderate to severe gingivitis (average GI score from 1.1 to 3) [30]. In our study, although there was a reduction in GI in both experimental groups after 21 days of use, the levels still remained within the mild GI range. A clinical trial conducted by Maher et al. evaluated the effectiveness of AgNPs and 0.12% CHX mouthwashes on *Streptococcus*, *Lactobacillus* spp., and *Candida albicans* in a cohort of Saudi children. The study reported excellent control of microbial counts, plaque index, and salivary pH in both groups. Notably, a significant difference was observed between CHX and AgNPs in terms of microbial load and dental plaque reduction, whereas no significant difference in salivary pH was found after 28 days of use. Although our findings are generally in agreement with Maher et al. regarding the comparable effectiveness of both mouthwashes, we did not observe a statistically significant difference between them in any of the measured parameters, except the QHI. This inconsistency may be attributed to variations in study design, including differences in participant randomization, sample characteristics, and methodological approaches [17].

The strengths of CHX, coupled with its side effects, continue to be a point of comparison for researchers seeking alternative compounds that may replace CHX. Our study results show that both CHX and AgNPs have nearly equivalent antibacterial effectiveness and can reduce gingival inflammation to a similar extent. However, in terms of plaque control, CHX still demonstrates superior effectiveness. This suggests that AgNPs could be used as a substitute for CHX when the primary goal is bacterial control and gingival inflammation. However, when plaque control is a priority, CHX should be preferred.

An important strength of our study is the random distribution of participants, with a double-blind study design and a sufficiently large sample size to ensure statistical significance. Furthermore, our research methodology for assessing the antibacterial effectiveness of mouthwash is broader than previous studies, which typically focused on only a few bacterial strains. On the other hand, the notable limitations of our study include the relatively short research duration and the inclusion of participants with generally good oral health. Therefore, future randomized controlled clinical trials with longer study periods and a larger, more diverse population may provide greater insights and value to this subject.

## Conclusions

The effectiveness of both 0.12% CHX and AgNPs mouthwashes in controlling gingivitis is primarily attributed to their antimicrobial properties, which reduce the oral bacterial load and improve salivary pH, both key factors in maintaining oral health. In this study, both formulations demonstrated significant effectiveness in reducing gingival inflammation and microbial counts. The 0.12% CHX mouthwash showed superior effectiveness in controlling dental plaque and continues to be considered the gold standard for the treatment of plaque-induced gingivitis. Meanwhile, AgNPs mouthwash also emerged as an effective alternative, offering satisfactory results in gingivitis control and oral microbial management.
